# The Relation of Fistula in Ano and Phthisis Pulmonalis

**Published:** 1888-02

**Authors:** Joseph M. Mathews

**Affiliations:** Louisville, Ky.; Professor of Principles and Practice of Surgery and Clinical Lecturer on Diseases of the Rectum, Kentucky School of Medicine; Visiting Surgeon, Louisville City Hospital, and Sts. Mary and Elizabeth Hospital, etc., etc.


VOL. IV. FEBRUARY, 1888. No. 12.

### Original Communications.

### THE RELATION OF FISTULA IN ANO AND PHTHISIS PULMONALIS.*

BY JOSEPH M. MATHEWS, M. D.,

Professor of Principles and Practice of Surgery and Clinical Lecturer on Diseases of the Rectum, Kentucky School of Medicine; Visiting Surgeon, Louisville City Hospital, and Sts. Mary and Elizabeth Hospital, etc., etc.

The common belief with the laity, and with many physicians, is that fistula in ano has some direct connection with the lungs, not only the diseased lung, but when it is in a perfectly healthy condition. This impression is wide-spread with patients suffering from rectal disease, and the questions propounded by them are sometimes really ludicrous.

I have been asked if the cure of piles would not result in consumption, and often have I had the objection preferred to curing a fistula that if the discharge was stopped, it would go to the lungs, and this, too, from persons who were of splendid physique

* Read before the Louisville Surgical Society.

and in perfect health, suffering only from a local sinus, caused likely by the passage of a fish-bone. I find, too, that patients have been prejudiced against the operation for the cure of fistula by some doctor who has warned them lest they have consumption as the consequence. Of course, to the learned physician this would be called pure nonsense, yet the prejudice exists, and we must use a sensible argument to refute it. Among the old authors the idea was prevalent that the discharge from a fistula *in the phthisical patient* had a modifying influence upon the disease.

There are many physicians yet who believe in this doctrine. This is commensurate with the belief in issues, setons, etc. From this early teaching many vagaries have resulted. Even the old writers, who knew but little pathology, did not believe, nor did they attempt to teach, that the curing of a fistula in a healthy person would result in phthisis, and it is very strange that in this day of research and pathological study that there are men who will, by an ill-advised word, consign a person to a life of disgust, if not of torture, by advising against an operation which will do no possible harm, but, on the contrary, relieve them of a life of suffering. It behooves us, then, as physicians, having the care of these cases, to look into the doctrine taught by the old masters in regard to this disease, and see if there be truth in it or not. It cannot be gainsaid that consumptives are frequently the subjects of fistula. These fistulæ may be dependent upon the disposition to phthisis or they may not. A person having diseased lungs may be just as liable to the other causes of fistula—*i. e.*, foreign bodies in the rectum, bruising, effect of cold, etc.—as persons who are perfectly healthy.

Is a fistula in ano in the consumptive patient a thing to be desired, for the reason advanced by the older writers, viz., that the discharge from the fistula modifies the disease of the lungs? If answered in the affirmative, I would ask if it would not be good surgery to *produce* an anal fistula in phthisical patients if they were so unfortunate as not to have one? The question at issue is: Shall we operate for fistula in ano in patients suffering from phthisis?

Before attempting to answer let us consider what some of the older writers have said on the subject.

In 1837, when Busch wrote his work on diseases of the rectum, he said: "It is very apparent that a great many fistulæ depend on disease of the lungs; therefore we should not operate on them, else their healing will give rise to an increase of the pulmonary disorder and curtail life." A few years before this, Brodie had said "no operation should be undertaken for fistula when phthisis is present, for one of two things will happen: either the sinus, although laid open, will not heal, or otherwise it *will* heal as usual, and the visceral disease will make more rapid progress, and the patient will die sooner than he would have done had he not fallen into the surgeon's hands." Sir Wm. Ferguson said that "the coincidence of fistula with disease of the lungs is often remarkable, and a surgeon would seldom be justified in interfering with a sinus under these circumstances." It seems that these really great men were given to theorizing in their day as many are given to the same thing now.

In these matter-of-fact times we want to know if theory is borne out by *clinical* facts. When Busch tells us "that it is very *apparent* that many fistulæ depend on diseased lungs, and that we should not operate because the healing will give rise to increased pulmonary disorder," he should have established this saying by clinical facts, or his statement is good for nothing. When Brodie affirms that under the same circumstances the wounds "will *not* heal, or if they *should* heal the patient will die sooner," he should have given us some statistics of operations to prove it. The mere statement of Sir Wm. Ferguson that "we are not justified in interfering with a sinus simply because the patient has a cavity in his lung," is not borne out by facts. Some of these writers say don't operate, because the wound *won't* heal; others that the wound *will* heal, and this is the danger. Certain I am that if I operate on any patient, under whatever circumstances, and the wound heals perfectly, I congratulate myself that I have done a good thing. The trouble in the consumptive patient is that the wounds *are* slow to heal, but not for the reasons usually given. But of this further on. Is there any degree of truth in the assertion of these gentlemen that we should not operate upon this class of patients? Are their reasons predicated upon facts? I imagine that this view has obtained credence principally because patients who have phthisis, complicated by fistula in ano, soon die of the lung trouble, and if perchance an operation has been done for fistula, the blame has been laid at the surgeon's door, albeit the life of the patient may have been prolonged, rather than shortened, by the operation. No good surgeon would risk his reputation by operating upon a patient who has rapid symptoms of advanced phthisis, as hectic, sweats, cough, emaciation, etc., but that there are many cases of consumption made worse by an existing fistula there can be no doubt. By curing the fistula the lung trouble will be benefited. There are several questions that should be considered before operating upon the phthisical patient for fistula:

1. Will the wound heal?

2. If the wound heals, will the patient be injured or benefited?

In answer to the first, there are several reasons for supposing that the wound would refuse to heal:

1st. When the lung trouble has advanced to the degree of emaciation, with cough, there is bad nutrition of all the tissues of the body; hence, the tissues around the rectum are embraced. The blood supply to the part is feeble and the proper return of the same by the veins is impeded. Under these circumstances the effort at repair would be poor. If cough exists, the succussion from the same would materially prevent the healing process. A more serious reason than these, however, is to be found in the condition of the parts operated on. The books are in the habit of giving three varieties of fistula—external, internal and complete. They forget to give the many varieties of these three, but make very plain (?) that a fistula is to be cured by the introduction of a director and the division of the main track. Any one who has operated many times for fistula knows how erroneous this is. Often, when only one small external opening presents, the cut and search will reveal many additional sinuses. Now, this is generally the case when fistula is found in the phthisical patient, not only many sinuses, but cavities of small calibre.

If any of these escape notice, the wound does not heal because of the continual flow from these. It would then become a question of importance whether the patient could bear that much cutting. The surgeon alone is to decide. It has been an observation of mine that wounds upon the consumptive heal readily. do not allude to fistulous wounds. The character of fistulæ may be very different from those described. I have seen many plain, uncomplicated cases of fistula in people who had consumption. A simple sinus, unprogressive fistula, which could not have resulted in any harm to divide, or would not have progressed if left. Then the character of fistulæ is very different, and it is upon this fact that I beg to differ from the learned men that I have quoted. Without regard to distinction they assert that *a fistula* must not be touched if the patient has phthisis. Patients are to be left to bear great pain and the annoyance of a continual discharge when they have a fistula of insignificant proportions, which could be easily cured. We will add more to the authority against the operation. Gross says: "All attempts at a radical cure are inadmissible when there are serious organic lesions in other parts of the body—especially the lungs. In such cases we cannot be too cautious, lest in arresting too suddenly a discharge, which has, perhaps, become habitual, we throw the onus on the more important organ and induce death prematurely."

Erichsen inclines to the idea that a fistula may act as a derivative in these cases, but says that in the early stages of phthisis an operation improves the patient's condition; then adds that an *issue* should be established in the arm or chest for a time.

These two opinions lead us to consider question second: If the wound heals, will the patient be injured or benefited?

We are to suppose, then, that the patient has vitality and nutrition sufficient for the wound to heal. Does the healing advance the phthisis? Both from a theoretical view and a practical demonstration I would answer in the negative. I have operated many times for fistula in the phthisical patient, and I have never had cause to regret it. Just as often, for reasons other than these, I have refused to operate. Is a fistula in ano a derivative for the lungs, as Erichsen intimates? If the principle of the doctrine of derivatives was correct, which I do not admit, this it appears to me to be the most far-fetched of all. A fistula in the rectum can have no bearing on the lung in a derivative way. The distance is too great, and there is no connection in an anatomical way—arterial, venous, capillary or nervous; hence, I cannot see what could be *derived* by it. A fistulous sinus is lined by a pyogenic membrane which discharges crude pus. The fistulous sinuses and cavities, which would exist as the result of the phthisical habit, would be filled with and discharge pus. In progressive fistula the tissue breaks down to pus by ulceration. Pus, in any form and in any quantity, is a waste. Then are we to suppose that by keeping up the waste we are to benefit an already enfeebled lung, or by stopping this waste we are, as Gross says, to hurry on the lung disease ? We cannot subscribe to this. If there was an over-abundance of some destructive material in the body, whose presence was working harm, and by a derivative, or what not, we could draw it away or *waste* it, then the proposition would appear reasonable; but here we are drawing from an already impoverished body. Add to this the mental anxiety that exists, and the removal of something disgusting and painful by an operation, then I am constrained to say that in many selected cases the operation should be done. Among those authorities that support this view are Allingham, Van Buren, Kelsey, Agnew, Bryant, Hamilton and George J. Cook.

### CONCLUSIONS.

1. In incipient phthisis the operation is always justifiable, other things being equal.

2. In the rapid, progressive fistula, an operation should be done to save tissue and prevent serious consequences.

3. If great cough exists it should militate against the operation.

4. If it can be determined that the sinus is single, and the discharge, inconvenience and pain not great, advise against the operation.

